# Editorial: Nanomaterials in food packaging

**DOI:** 10.3389/fnut.2022.1083185

**Published:** 2022-11-15

**Authors:** Coralia V. Garcia

**Affiliations:** Department of Food Science & Technology, Keimyung University, Daegu, South Korea

**Keywords:** nanomaterials, nanoparticles, food packaging, nanocomposites, active packaging

Food packaging is indispensable for modern food production, and even though advances like multilayered packaging have resulted in enhanced barrier properties, improvements resulting in packaging that is transparent, light and biodegradable, or easy to recycle are still needed (1).

Nanocomposites can be produced by combining nanomaterials with polymers. The nanomaterials act as a reinforcement and can also enhance the mechanical, barrier, and thermal properties of the composites, resulting in novel packaging materials with improved properties. In addition, active packaging with antimicrobial, antioxidant, and other effects can be developed using nanomaterials (2). In addition to the well-known silver nanoparticles and nanoclay, nanomaterials used in packaging include nano-metal oxides (3), nanocellulose (4), and halloysite nanotubes and essential oils (5). These nanomaterials can confer various activities to the packaging composites including antioxidant (e.g., essential oils), antimicrobial (e.g., nanosilver), ethylene scavenging (e.g., nano-KMnO4), and oxygen scavenging (e.g., Pd nanoparticles) activities as described in a recent review article (6).

This Research Topic aimed at collecting papers that demonstrate the application of nanomaterials to polymers to fabricate nanocomposites that can be used in food packaging. Furthermore, special emphasis was given to active packaging that exhibited a substantial improvement over conventional packaging materials.

In this special e-collection, four papers covered this aspect.

Kwon et al. produced kraft fiber-silver nanoparticle composite sheets with antimicrobial activity against *E. coli*. Even though cellulose sheets like these show low water barrier properties, their usefulness is in paper packaging applications where antibacterial effects are desirable. Another type of antimicrobial nanomaterial was reported by Muñoz-Shugulí et al., who developed β-cyclodextrin complexes incorporating allyl isothiocyanate. These complexes demonstrated antifungal activity against *Botrytis cinerea* as well as humidity-responsive changes, and could potentially be used as antifungal agents in food packaging.

One of the most important qualities of food packaging is its barrier properties. Incorporating nanomaterials generally enhances the moisture and gas barrier properties of the resulting composites. The dispersion of nanoparticles in the polymer matrix hinders the polymer chain mobility and creates tortuous paths through which the water and gas molecules must travel, thus reducing water permeability and gas absorption (6). Schiessl et al. developed high-barrier nanocomposite coatings based on polyvinyl alcohol (PVA) and montmorillonite (MMT). These researchers used the “nanolacquer” technique to produce nanocomposites incorporating platelet-shaped MMT. The nanocomposites exhibited enhanced gas barrier properties, which were ascribed to the tortuous path effect. Nanocomposites such as this could be used in the production of packaging with increased oxygen barrier properties.

The development of ethylene scavengers using nanomaterials was reported by Rudra et al., who described nanomats that encapsulated palladium chloride, a compound that exhibits ethylene-scavenging properties. The nanomats were placed into boxes containing sapota fruits, resulting in enhanced quality indices during storage. The fruits packaged with the nanomats were firmer and showed lower weight loss, demonstrating the effectiveness of this strategy.

In summary, the studies included in this collection provide new data on the development of composites using nanomaterials and the resulting improvements in the barrier properties of potential new packaging materials. In addition, materials that could be used as active packaging with antimicrobial and ethylene scavenging activities were reported. The papers included in this collection demonstrate that nanomaterials show great potential for the development of novel packaging materials, and may serve as an inspiration for future studies.

## Author contributions

The author confirms being the sole contributor of this work and has approved it for publication.

## Conflict of interest

The author declares that the research was conducted in the absence of any commercial or financial relationships that could be construed as a potential conflict of interest.

## Publisher's note

All claims expressed in this article are solely those of the authors and do not necessarily represent those of their affiliated organizations, or those of the publisher, the editors and the reviewers. Any product that may be evaluated in this article, or claim that may be made by its manufacturer, is not guaranteed or endorsed by the publisher.
